# Supplementary material for: Bringing uncertainty quantification to the extreme-edge with memristor-based Bayesian neural networks
Source: Nat Commun. 2023 Nov 20;14:7530. doi: 10.1038/s41467-023-43317-9 (PMC10661910; doi:10.1038/s41467-023-43317-9)
Supplement: Supplementary file 1 — Supplementary Information [file 41467_2023_43317_MOESM1_ESM.pdf]

Supplementary information: Bringing uncertainty quantification to the extreme-edge with memristor-based Bayesian neural networks

Djohan Bonnet[1,2*], Tifenn Hirtzlin[1], Atreya Majumdar[2], Thomas Dalgaty[3], Eduardo Esmanhotto[1], Valentina Meli[1], Niccolo Castellani[1], Simon Martin[1], Jean-François Nodin[1], Guillaume Bourgeois[1], Jean-Michel Portal[4], Damien Querlioz[2*], and Elisa Vianello[1*]

[1]Université Grenoble Alpes, CEA, LETI, Grenoble, France
[2]Université Paris-Saclay, CNRS, Centre de Nanosciences et de Nanotechnologies, Palaiseau, France
[3]Université Grenoble Alpes, CEA, LIST, Grenoble, France
[4]Aix-Marseille Université, CNRS, Institut Matériaux Microélectronique Nanosciences de Provence, Marseille, France
[*]djohan.bonnet@cea.fr, damien.querlioz@c2n.upsaclay,fr, elisa.vianello@cea.fr

Contents

Supplementary Note 1: Quantifying the performance of epistemic and aleatoric uncertainty estimation 1
Supplementary Note 2: Experimental validation of the Bayesian neural network behavioral inference simulator 3
Supplementary Note 3: Evaluating cycle-to-cycle variability in memristor and PCM programming 3
Supplementary Note 4: Measurements of the conductance drift in phase-change memory devices 5
Supplementary Note 5: Measurements of the conductance relaxation in memristor devices 7
Supplementary Note 6: Impact of relaxation/drift on Bayesian Neural Network 7
Supplementary Note 7: Impact of number of devices per sample on Bayesian Neural Network 8
Supplementary Note 8: Impact of iterative programming on Bayesian Neural Network 9
Supplementary Note 9: Impact of technological loss on Bayesian Neural Network 10
Supplementary Note 10: Transfer of a Bayesian neural network trained with Markov Chain Monte Carlo sampling to a resistive memory-based inference hardware 11
Supplementary Note 11: Method validation on the MNIST dataset 12
Supplementary Note 12: Method validation on CIFAR datasets 13
Supplementary Note 13: In-memory computing chip 14
Supplementary Note 14: Benchmark 15
Supplementary Note 15: Inference energy consumption estimates 17
Supplementary Note 16: Flowchart of the Bayesian neural network inference experiment 17
Supplementary References 20

Supplementary Note 1: Quantifying the performance of epistemic and aleatoric uncertainty estimation

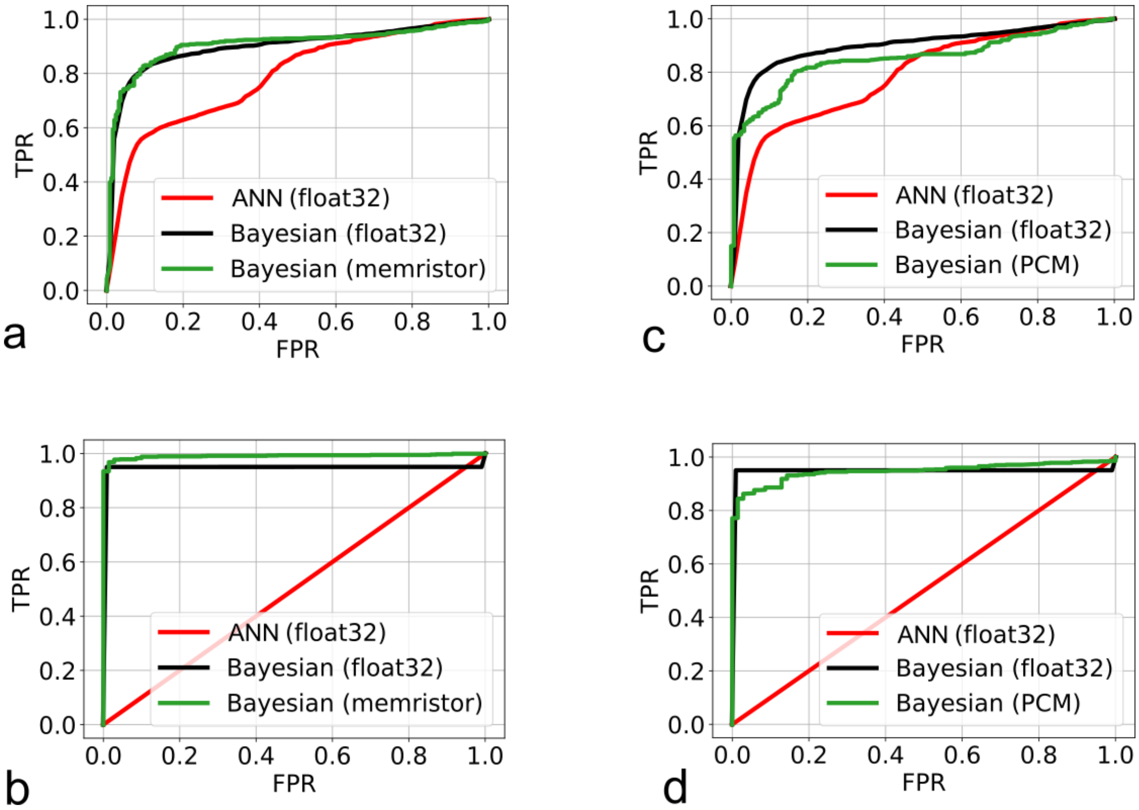

Supplementary Figure 1. Receiver Operating Characteristic (ROC). a ROC curve corresponding to the differentiation between correct prediction and incorrect prediction, based on aleatoric uncertainty measured on the proposed memristor-based Bayesian neural network (green) and simulated (weights stored as $float32$ real numbers) for Bayesian (black) and conventional artificial neural network (red) with a fully connected neural network of size (32,16,9). **b** ROC curve corresponding to the differentiation between known and unknown data, based on epistemic uncertainty measured on the proposed memristor-based Bayesian neural network (green) and simulated (weights stored as $float32$ real numbers) for Bayesian (black) and conventional artificial neural network with the same architecture (red). **c** ROC curve corresponding to the differentiation between correct prediction and incorrect prediction, based on aleatoric uncertainty measured on the proposed PCM-based Bayesian neural network (green) and simulated (weights stored as $float32$ real numbers) for Bayesian (black) and conventional artificial neural network with the same architecture (red). **d** ROC curve corresponding to the differentiation between known and unknown data, based on epistemic uncertainty measured on the proposed PCM-based Bayesian neural network (green) and simulated (weights stored as $float32$ real numbers) for Bayesian (black) and conventional artificial neural network with the same architecture (red).

In our study, we emphasize the importance of measuring the performance in evaluating both aleatoric and epistemic uncertainty. To assess these performances, we used receiver operating characteristic (ROC) curves, a widely used metric for diagnostic ability, obtained by plotting the true positive rate as a function of the false positive rate for various threshold settings. A perfect classifier would yield the (0, 1) point, i.e., an area under the curve (AUC) of one, corresponding to no false negatives and no false positives. The ROC curve of a random classifier approaches the diagonal line, i.e., an area under the curve of 0.5. This Note details how we employed these curves in our study, and shows some ROC curves used to obtain Table 1 in the main text.

Supplementary Fig. 1a shows the ROC curve corresponding to the differentiation between correct predictions and incorrect predictions, based on aleatoric uncertainty, for our memristor experiment, a purely software version of the Bayesian neural network programmed in our experiment, and a conventional neural network with the same architecture (see Methods of the main text). Our experiment leads to a ROC curve close to the software Bayesian neural network, showing the high quality of our transfer. In this case, it even slightly outperforms the software version (area under the curve of 0.91 vs. 0.90). In this typical

case, the conventional neural network has an inferior area under the curve (0.79), highlighting the tendency of such networks for overconfidence. We should remark that an area under the curve of one is impossible for this graph, because predictions with low aleatoric certainty are sometimes correct.

Supplementary Fig. 1b shows the ROC curve corresponding to the differentiation between known and unknown data, based on epistemic uncertainty (see Methods of the main text). Our experiment performs particularly well, with an area under the curve of 0.99, close to the perfect value of one, and which slightly outperforms the one obtained by the software (0.95) Bayesian neural network in this typical case. Still, the best software neural networks match the accuracy of our experiment (see the Results section of the main body text). As the epistemic uncertainty cannot be computed for a deterministic neural network, we have represented its ROC curve as a diagonal line (x=y) for reference.

Supplementary Figs. 1c,d show similar ROC curves with simulations of Bayesian neural networks employing phase change memories.

All results presented in the main text in Fig. 5h and in Table 1 are obtained by measuring the area under the curve of such ROC curves.

Supplementary Note 2: Experimental validation of the Bayesian neural network behavioral inference simulator

We developed a custom behavioral simulation of our experiment in Python, implementing Bayesian neural network inference after training with the proposed variational inference technique, including the "technological loss" term. Our simulator reproduces the memory device programming process by sampling their resistance values with normal distributions calibrated on measured data (Fig. 3 in the main paper). To do so each trained synaptic weight is assigned to the nearest experimental value, determined using the Kullback-Leibler divergence as the metric. This step emulates the transfer to a hardware implementation. We included models for both filamentary memristors and phase change memories crossbar arrays.

Following our experiments, the simulator samples the M real values programmed into the M distinct memory arrays. It then reproduces the inference process using Ohm's law and Kirchoff's laws. We repeated this simulation 50 times, and Supplementary Figure 2 reports the average performance obtained from these 50 iterations. The error bars on the bar plots represent one standard deviation. Supplementary Fig. 2 compares the results of the simulator with the experimental ones reported in the main Article, obtained on the Bayesian neural network composed of 75 physical crossbar arrays of filamentary-based memristors to classify arrhythmia in ECG recordings. This Supplementary Figure shows the accuracy, and the capability of modeling epistemic uncertainty, and aleatoric uncertainty (reported using areas under the curve, see main Article) as a function of the number of filamentary memristor devices per synapse. It is observed that the behavioral simulator shows a good correspondence with the experimental results; it can therefore serve as a useful tool in testing alternative approaches, e.g., other technologies.

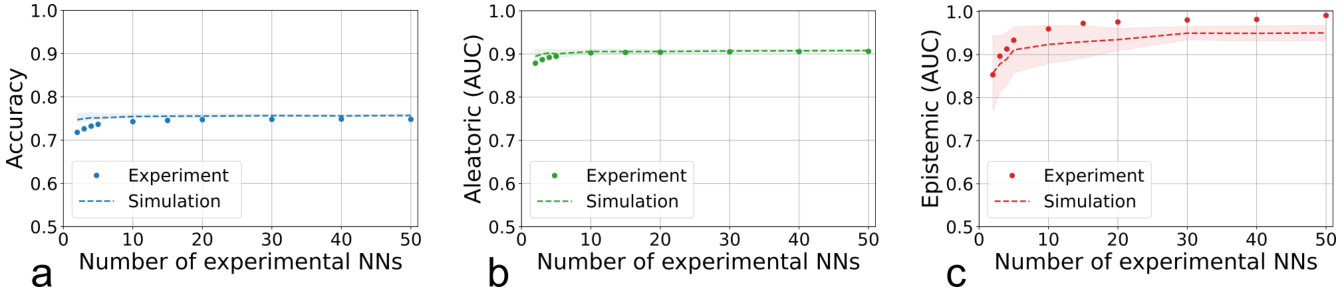

Supplementary Figure 2. Experimental (symbols) and simulated (dashed line) accuracy **a**, aleatoric uncertainty **b**, and epistemic uncertainty **c** (calculated as the area of the ROC curves) as a function of the number of filamentary memristor devices per synapse.

Supplementary Note 3: Evaluating cycle to cycle variability in memristor and phase change memory programming

This Note aims at comparing cycle-to-cycle and device-to-device variability, for filamentary memristors and phase change memories. For both technologies, we conducted additional electrical characterization by performing 1,000 cycles on an array of 1,000 distinct devices for different programming conditions.

First, we check that device-to-device variability is stable when programming the array multiple times (see supplementary Figures 3a and 3b, for memristors and phase change memories, respectively). Distinct clusters of data points represent different programming conditions. Each data point represents the mean and standard deviation of a Gaussian distribution obtained by programming 1,000 distinct devices, with different points representing the 1,000 cycles. We see that the cycle-to-cycle variability does not affect the mean and standard deviation of the Gaussian distribution obtained from exploiting cell-to-cell variation across all studied conditions.

To understand this result further, Supplementary Figures 3c and 3d compare the probability density of 1,000 distinct devices programmed once (device-to-device variability) with the probability density of one device cycled 1,000 times (cycle-to-cycle variability) for memristors and PCM technologies, respectively. For each technology, we have presented the distributions obtained using the programming conditions corresponding to the smallest and largest mean conductance values, as well as an intermediate distribution. Both sources of variability exhibit an equivalent impact on conductance variability across different programming conditions.

The reason for the apparent equivalence between device-to-device and cycle-to-device variability differs for the two types of devices. For memristors, the conductance depends on the shape of the filament, which varies cycle-to-cycle and depends weakly on the specific properties of a particular structure. In the case of phase change memories, by contrast, the equivalence originates solely due to the use of iterative programming.

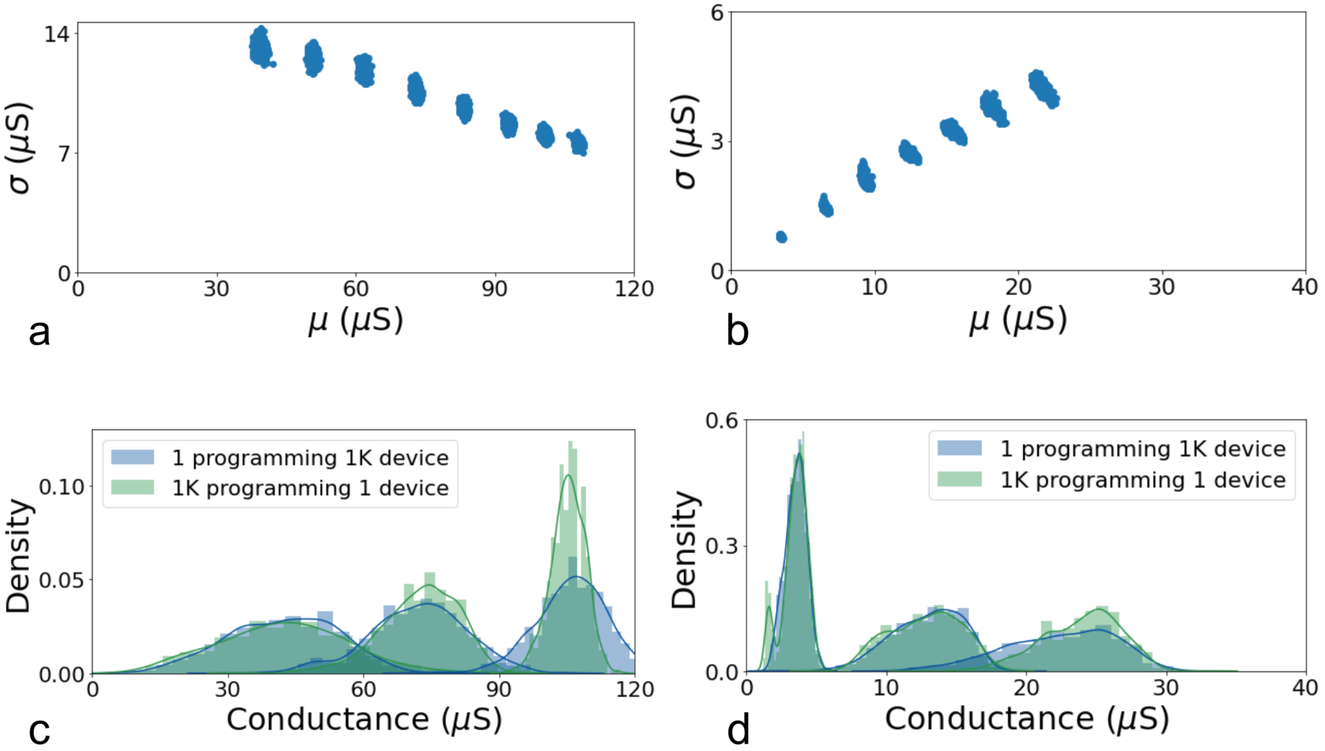

Supplementary Figure 3. Impact of cycle-to-cycle variability on cell-to-cell variations for memristors and PCMs. **a** Scatter plot illustrating the influence of cycle-to-cycle variability on cell-to-cell variation in memristors for different programming conditions. Different clusters of data points represent distinct programming conditions. Each data point reflects the mean and standard deviation of a Gaussian distribution obtained by programming 1,000 distinct devices, with varying points representing 1,000 cycles per programming condition. **b** Scatter plot illustrating the effect of cycle-to-cycle variability on cell-to-cell variation in phase change memory for different programming conditions. **c** Probability density of 1,000 cells programmed under the same conditions, depicting device-to-device variability (blue), compared with the probability density of one device programmed 1,000 times (green) for memristor technology. The experiment has been repeated for three different programming conditions corresponding to the smallest and largest mean conductance values in **a**, as well as an intermediate distribution. **d** Probability density of 1,000 cells programmed under the same conditions, illustrating device-to-device variability (blue), compared with the probability density of one device programmed 1,000 times (green) for PCM technology. The experiment has been repeated for three different programming conditions corresponding to the smallest and largest mean conductance values in **b**, as well as an intermediate distribution.

Supplementary Note 4: Measurements of the conductance drift in phase-change memory devices

We have seen in the main article that the variability in phase-change memories (PCM) can be exploited to implement normal distributions with different mean values μ and standard deviations σ (main article, Fig. 4). However, in PCMs, amorphous regions of the chalcogenide material naturally undergo structural relaxation, and the conductance tends to decrease with time[1,2]. This ubiquitous phenomenon in PCMs, known as conductance drift, affects the distribution of programmed states into PCMs.

To study the impact of the conductance drift on the technologically plausible domain of the normal distributions, Γ_{PCM}, we measured the distributions of 16,384 PCM devices programmed in eight conductance levels (2,048 devices per level), over two weeks. Supplementary Fig. 4 shows the 64 different Bayesian synapse states (μ, σ) that can be obtained by combining these eight states: they cover the whole technologically plausible domain of the normal distributions, Γ_{PCM}. In the same Supplementary Figure, we plot the evolution of the mean and standard deviation of the synapses in each state after one hour, one day, and two weeks. We see that the drift causes a compression of the Γ_{PCM} domain towards lower conductance values. After a day, the effect of this compression becomes negligible, consistently with the known power law behavior of PCM drift.

This result leads to a natural solution to program drift-immune Bayesian neural networks with PCMs. When training a PCM Bayesian neural network, the "technological loss term" should not be based on the technologically plausible domain of the normal distributions Γ_{PCM} obtained right after programming, but the one after drift. In our studies, we use the domain

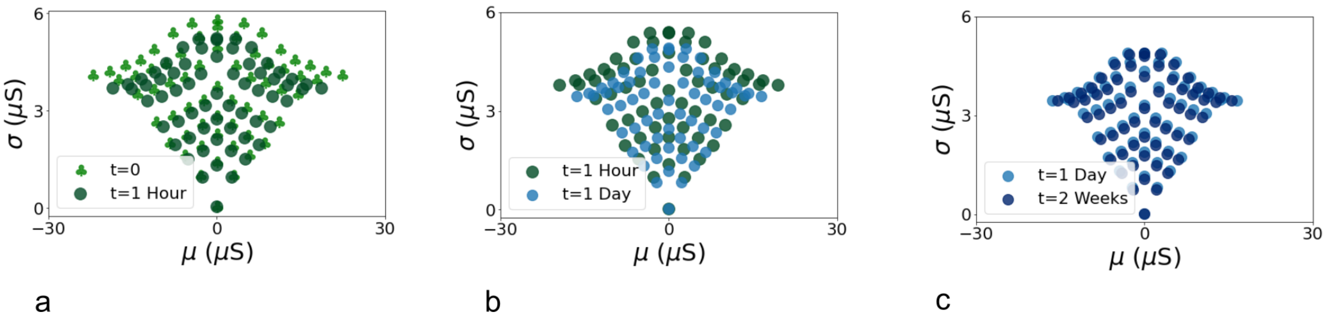

Supplementary Figure 4. Domain of the normal distributions (Γ_{PCM}) measured at different times after programming. **(a)** Right after programming and at t=1 hour, **(b)** t=1 hour and t=1 day, **(c)** t=1 day and t=2 weeks.

reached after one day of drift.

Supplementary Note 5: Measurements of the conductance relaxation in memristor devices

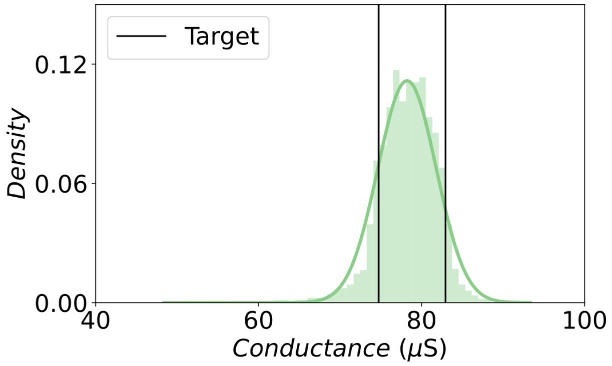

Supplementary Figure 5. Probability density of 1,024 memristors after short-term relaxation (6 seconds after iterative programming) and the target conductance values used during the iterative programming.

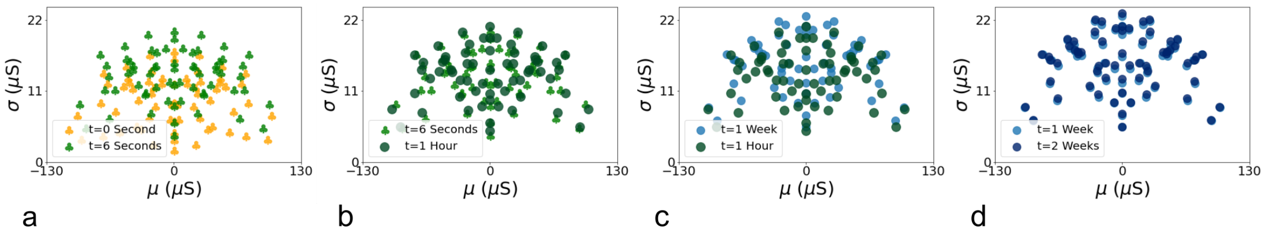

Supplementary Figure 6. Domain of the normal distributions ($\Gamma_{memristor}$) measured at different times after programming. **a** at t=0 second and right after short-term relaxation at t=6 seconds, **b** right after short-term relaxation at t=6 seconds and at t=1 hour, **c** at t=1 hour and at t=1 week, **d** at t=1 week and at t=2 weeks.

To mitigate the conductance variability, we adopted an iterative programming strategy (Figure 3b in the main). The memristor is programmed multiple times until its resistance reaches the target value. However, as shown in Figure 5, memristors suffer from conductance relaxation within a few seconds after programming, causing the conductance distribution to spread towards both higher and lower values[3]. Consequently, the technological loss term is computed based on the technologically plausible domain of normal distributions obtained after 6 seconds (i.e. after relaxation). This conductance spread continues at a slower rate over longer time scales, as demonstrated in Figure 6. This phenomenon was also previously observed in our work[4]. However, as Supplementary Note 7 demonstrates, this long-term relaxation has only a minimal impact on the neural network's performance.

Figure 5 compares the probability density of 1,024 memristors initially ($t = 0s$) and after relaxation (six seconds after iterative programming) with the target conductance values. Additionally, Figure 6 shows the technologically plausible domain of the normal distributions, $\Gamma_{memristor}$, over a span of two weeks. These results confirm that the mean value of conductance distributions remain stable after the initial six seconds, and the standard deviations only slightly increase during the first week. For this reason, we used the conductance distributions after relaxation as experimental data, denoted as $\theta_{exp} = (\mu_{exp}, \sigma_{exp})$, to define the technological loss term (Eq. 9 in the main text) to ensure a stable programming of the Bayesian neural network. Supplementary Figure 7 confirms that the increase in standard deviation is not strong enough to affect the performances.

Supplementary Note 6: Impact of relaxation/drift on Bayesian Neural Networks

To study the evolution of network performance over time, we conducted simulations using calibrated experimental data from Supplementary Figure 6 for memristors and Supplementary Figure 4 for phase change memory. Since the effects of phase change memory drift (Supplementary Note 4) and memristor conductance relaxation (Supplementary Note 5) become negligible after one day and after six seconds, respectively, the technological loss term is based on the technologically plausible domain of

normal distributions obtained after one day for phase change memory and after six seconds for memristors. The simulations involve two separate training processes, one for memristors and another for phase change memory.

To generate Supplementary Figure 7, we conducted simulations for our reference arrhythmia classification task using a fully connected Bayesian neural network (32,16,9) with M=50 samples, as described in the Methods section of the main article. The simulations were performed for both memristor and phase change memory implementations. We utilized the simulator validated in Supplementary Note 2. In this simulator, the synaptic weights are modeled based on Gaussian distributions calibrated on experimental measurements. Each trained synaptic weight is assigned to the nearest experimental value, determined using the Kullback-Leibler divergence as the metric. This step emulates the transfer to a hardware implementation. To evaluate the performances over time, we performed this transfer using distributions measured at different times. Following the transfer, we assessed the performance of the Bayesian neural network. This simulation was repeated 50 times, and the average performance at different times, obtained from these 50 iterations, is presented in Supplementary Figure 7. The error bars on the bar plots represent one standard deviation.

Importantly, our results demonstrate that the Bayesian Neural Network exhibits remarkable resilience to challenges posed by conductance relaxation and drift.

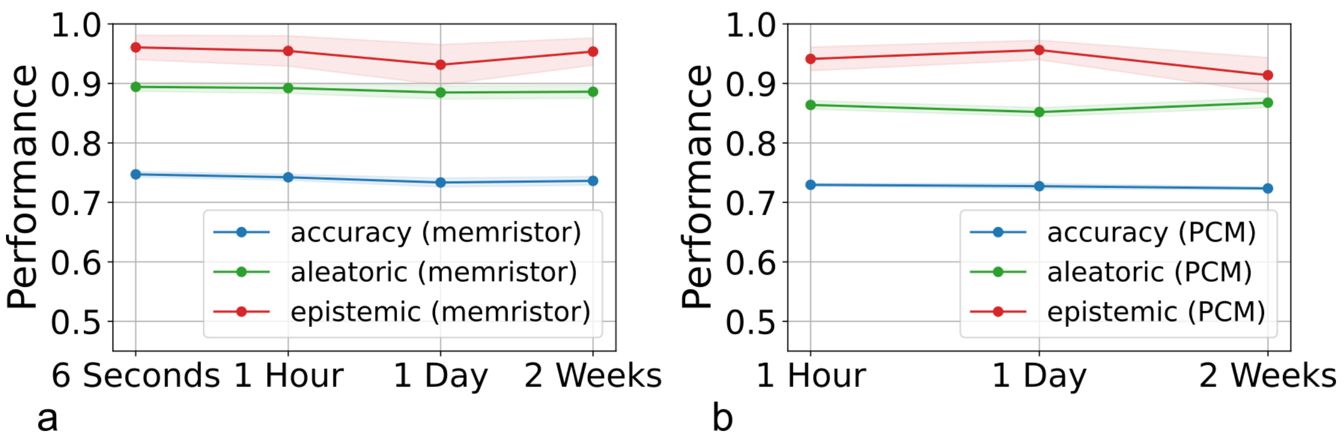

Supplementary Figure 7. Network performances in terms of accuracy and uncertainty estimation over time for Bayesian neural network based on memristors **a** and PCMs **b**.

Supplementary Note 7: Impact of number of devices per sample on Bayesian Neural Network

In our main paper, we employed a strategy of using two cells per sample, which we discovered to be a favorable balance between the number of devices utilized and overall performance. In this Supplementary Note, we provide further explanations and insights into why we made this choice.

Supplementary Figure 8a illustrates the domain of normal distributions obtained using one (blue), two (green), and four (yellow) devices per sample of a Bayesian probabilistic weight. Increasing the number of devices per sample expands the attainable size of the domain of normal distributions in experimental settings, which is favorable.

Supplementary Figure 8b shows the performance that we would obtain on our reference arrhythmia classification task in terms of raw accuracy and uncertainty evaluation, using one, two, or four devices (i.e., two pairs) per weight sample. These results employ the simulator of our experiment (validated in Supplementary Note 2) and a Bayesian neural network with M=50 samples.

The results of Supplementary Figure 8b confirm that a wide range of possibilities for σ and μ, obtained by increasing the number of devices per sample, enhances both neural network accuracy and uncertainty estimation. A broad spectrum of possibilities for μ is indeed crucial for achieving high accuracy, while a diverse range of possibilities for σ is necessary for accurate uncertainty estimation. A small σ value ensures minimal output variability for well-known situations, and a large σ value provides output variability for unknown data, thereby allowing uncertainty estimation.

Regarding the performances, Supplementary Figure 8b shows a modest increase of less than one percentage point in both accuracy and epistemic and aleatoric uncertainty using two pairs of memristors per sample. Considering the resource implications of doubling the number of memristors, we made the deliberate choice to conduct the experiment using only one pair.

Conversely, Supplementary Figure 8b shows that using a single device per weight sample further leads to an important loss of accuracy.

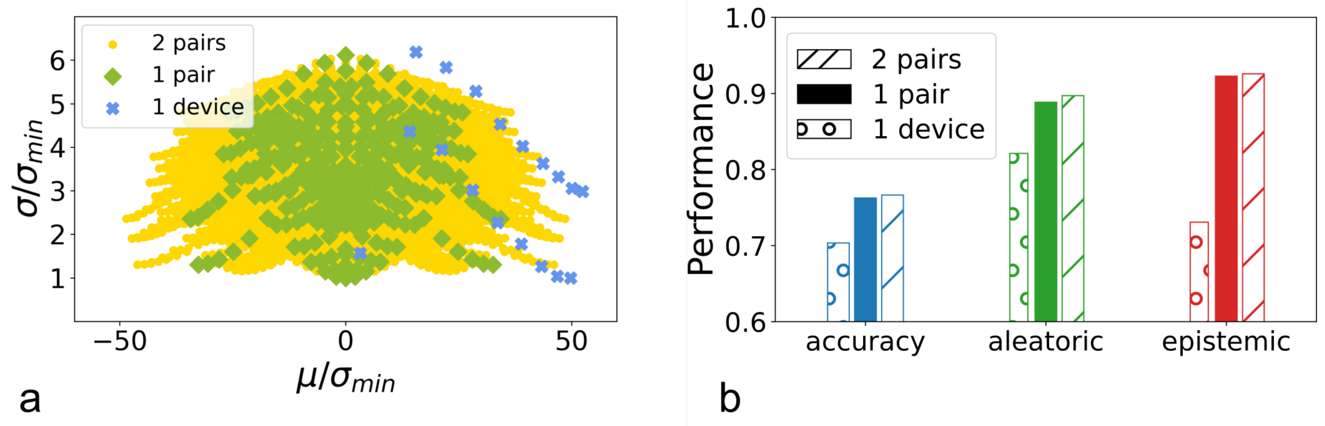

Supplementary Figure 8. Impact of the number of memristors per sample of a Bayesian probabilistic weight a Domain of the normal distribution obtained experimentally by storing samples on one (blue), two (green), and four (yellow) memristors. μ and σ are normalized to the minimum achievable standard deviation, σ_{min}, obtained with different experimental conditions.**b** Network performances obtained by storing samples on one, two, and four memristors. The performances are evaluated for ten training runs. The training process was repeated ten times, and each inference was repeated 50 times with $M = 50$ samples. The final result represents the average performance obtained from the 500 inferences.

Supplementary Note 8: Impact of iterative programming on Bayesian Neural Network

In the main text, we highlighted that our approach incorporates not only classical set programming and reset but also employs iterative programming with a program-and-verify scheme. This programming method introduces additional complexity and thus requires justification based on its impact on the performances of the Bayesian neural network.

To visualize the effect of iterative programming in the case of filamentary memristors, Supplementary Figure 9a illustrates the domain of normal distributions obtained with (green) and without (blue) the use of iterative programming. This Figure is plotted using the same methodology as Fig. 3e of the main article. The inclusion of iterative programming expands the range of achievable σ values in experimental results. Note that iterative programming was mandatory for PCM, as the set distributions were incredibly spread in the high conductance states.

To generate Supplementary Figure 9b, we simulated our reference arrhythmia classification task, employing a fully connected Bayesian neural network (32,16,9) with $M = 10$ samples (see Methods of the main article), with and without the use of iterative programming. For this, we used the simulator validated in Supplementary Note 2. The training process was repeated ten times, and each inference was repeated 50 times. The final result represents the average performance obtained from the 500 inferences.

The results of Supplementary Figure 9b confirm that the broad spectrum of possibilities for σ provided by iterative programming improves the estimation of aleatoric uncertainty by three percentage points. Synapses with a small σ value are important to ensure minimal output variability for non-ambiguous situations. The accuracy and epistemic uncertainty performances experience smaller improvements, with gains of two and one percentage points respectively. Therefore, the use of iterative programming enhances accuracy and uncertainty evaluation, but the domain without iterative programming already accommodates the requirements of our application and the associated metrics. For applications with higher complexity, we

anticipate that the expansion of the domain will become increasingly necessary.

For phase change memory, by contrast, iterative programming is fundamental to programming the devices and is used in all cases.

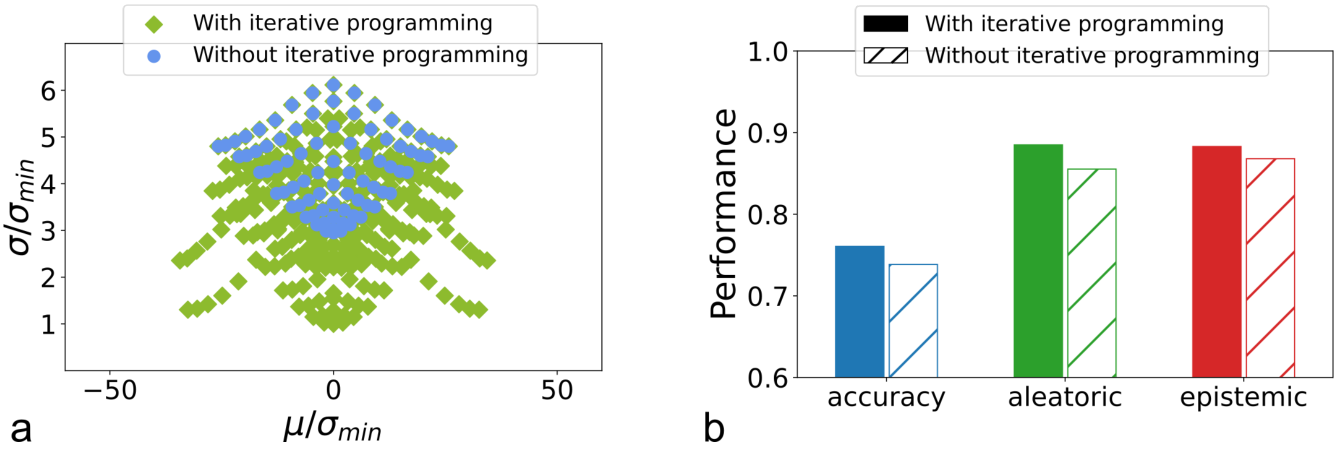

Supplementary Figure 9. Impact of iterative programming. a Domain of the normal distribution obtained experimentally by storing samples without (blue) and with (green) iterative programming. μ and σ are normalized to the minimum achievable standard deviation, σ_{min}, obtained with different experimental conditions. **b** Network performances obtained by storing samples without and with iterative programming. The training process was repeated ten times, and each inference was repeated 50 times with $M = 10$ samples. The final result represents the average performance obtained from the 500 inferences.

Supplementary Note 9: Impact of technological loss on Bayesian Neural Network

The device physics of both filamentary memristors and phase change memory imposes limitations on the range of Bayesian weights that can be stored (see Figure 3e in the main text). These limitations result in significant performance losses when attempting to transfer a model trained off-chip using the classical Bayes By Backprop method onto hardware chips. The issue arises because the training algorithm may require a weight that cannot be implemented in hardware. To address this challenge, in this work, we proposed a hardware-calibrated training method that considers the technological constraints associated with memristor or phase change memory technology. By introducing a technological loss term, this approach restricts the domain of achievable weights to those that can be experimentally realized with the given technology. In this Note, we show the impact of using this technological loss.

To generate Supplementary Figure 10b, we conducted simulations for our reference arrhythmia classification task using a fully connected Bayesian neural network (32,16,9) with M=50 samples. We compared the performance of models trained with and without the inclusion of technological loss. These simulations were performed using the validated simulator described in Supplementary Note 2. The training process was repeated ten times, and each inference was repeated 50 times. The final result represents the average performance obtained from the 500 inferences.

Supplementary Figures 10a and b compare the network accuracy and uncertainty estimation obtained by training a Bayesian neural network with and without using the technological loss. The results confirm that the technological loss allows for important improvement in the network performances in terms of both accuracy and uncertainty estimation. We can also observe that without the technological loss, the degradation is greater for phase change memories than for filamentary results. This result was predictable as the technological constraints are greater for phase change memories than filamentary memristors (see Fig. 3 of the main article).

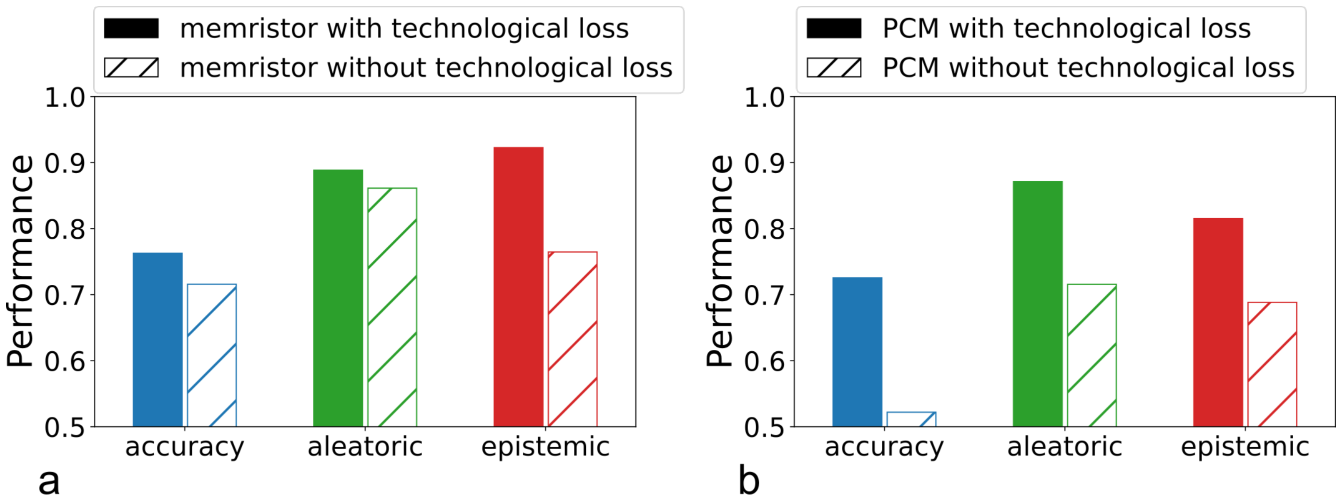

Supplementary Figure 10. **a** Network performances obtained on a memristor-based Bayesian network trained with the popular Bayes By Backprop method and the novel hardware calibrated training algorithm based on the technology loss. **b** Network performances obtained on a PCM-based Bayesian network trained with the popular Bayes By Backprop method and the novel hardware-calibrated training algorithm based on the technology loss. The training process was repeated ten times, and each inference was repeated 50 times with $M = 10$ samples. The final result represents the average performance obtained from the 500 inferences.

Supplementary Note 10: Transfer of a Bayesian neural network trained with Markov Chain Monte Carlo sampling to a resistive memory-based inference hardware

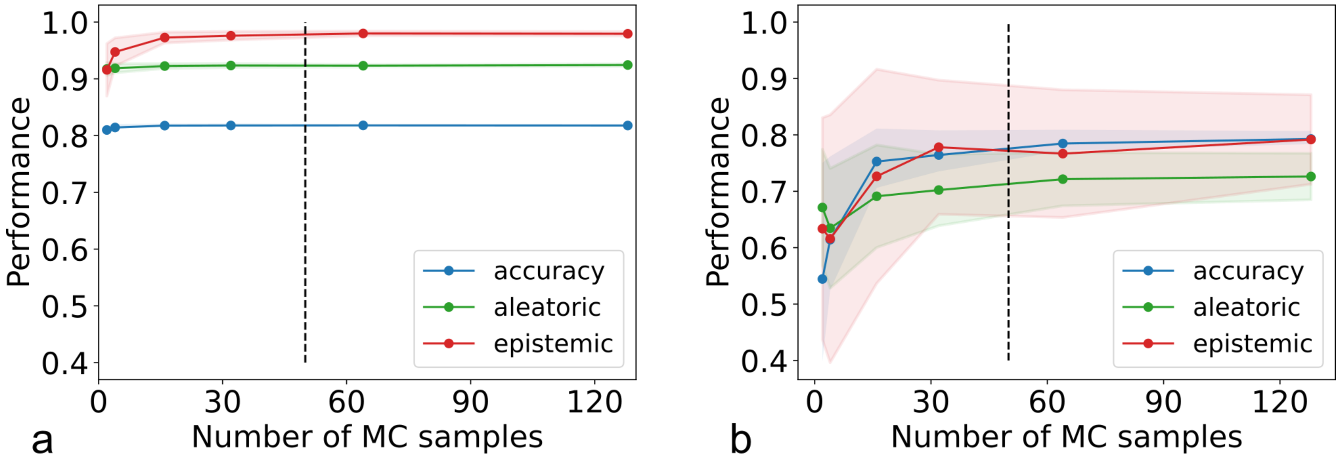

Supplementary Figure 11. Simulated accuracy, epistemic uncertainty, and aleatoric uncertainty (calculated as the area of the ROC curves) before **a** and after **b** the transfer on filamentary memristor crossbar arrays as a function of the number of MC samples (i.e. number of memristor devices per synapse).

Variational Inference and Markov Chain Monte Carlo (MCMC) sampling are two popular techniques for training Bayesian neural networks. They both aim at approximating the posterior $p(\boldsymbol{\Omega}|\boldsymbol{D})$, where $\boldsymbol{\Omega}$ represents the synaptic weights, and $\boldsymbol{D}$ is the training data. Our method for realizing memristor-based Bayesian neural networks uses variational inference, whereas the prior simulation works of[5] proposed using MCMC. In this Note, we compare these two techniques.

Markov Chain Monte Carlo provides a collection of models, each with a specific weight value for each synapse. Models are proposed based on a sampling procedure, and then either accepted or rejected. After a sufficiently large number of iterations, the accepted samples describe, together, an approximation of the posterior distribution, $p(\boldsymbol{\Omega}|\boldsymbol{D})$.

When using MCMC, the values of the synaptic weights from different models should not be mixed: MCMC models the whole distribution $p(\boldsymbol{\Omega}|\boldsymbol{D})$, including the correlation between weight values. Variational inference, by contrast, simplifies the

problem. Each synapse is assumed to be statistically independent, and its probability distribution is supposed to be a normal law with a mean value μ and a standard deviation σ. The goal of the learning process is to identify the μ and σ values for all synapses, which is achieved by backpropagation over an appropriate loss function (see equation (1), main Article).

We trained a Bayesian neural network to classify ECG diagnoses beats, in the same conditions as in the main article, using MCMC. Supplementary Fig. 11a shows the accuracy and the quality of the estimations of epistemic uncertainty and aleatoric uncertainty (evaluated by means of area under the curve of the two ROC curves introduced in the main Article, Figs. 5f, and g) of a simulated Bayesian neural network, as a function of the number of considered MCMC samples. The maximum accuracy is 82%, and the maximum area under curves corresponding to aleatoric and epistemic uncertainty are 0.92 and 0.98. These numbers slightly outperform those obtained with variational inference in the main article (79%, 0.90, and 0.95, see Table I, main article). This result illustrates the benefits that MCMC models correlation (covariance) between values of the synapses.

However, this covariance between synapse values is very hard to maintain after transfer to memristors, due to memristor imperfections. We simulated the transfer of the MCMC-trained Bayesian neural network to our memristor using the technique proposed in[5], which is specifically designed to keep the covariance despite device imperfection. We plotted the resulting accuracy and evaluation of aleatoric and epistemic uncertainty in Supplementary Fig. 11b. The accuracy is close to the one before transfer, 79%, but the capability to assess both aleatoric and epistemic uncertainty is strongly degraded by the transfer operation (area under the curves of 0.73 and 0.79, respectively). This degradation originates because of the noise when programming the memristor, making it highly challenging to keep the correlation between synaptic values exactly.

This result contrasts with the ones in the main Article using variational inference, where it is seen that the Bayesian neural network before and after transfer have similar accuracy and similar capability to model uncertainty (see Table I, main article). The reason for this robustness is straightforward. Because variational inference assumes independence between the synapses, they are programmed independently, following their probability distribution. And because our technique matches the device probability distribution with the one of the Bayesian neural network, programming noise does not degrade the operation of the network: noise is in fact exploited to perform the sampling operation of the Bayesian neural network.

Additionally, variational inference is known to scale much better than MCMC[6]. To solve more difficult tasks involving a higher number of memristors, variational inference will be the only approach capable to train the network.

Supplementary Note 11: Method validation on the MNIST dataset

In the main body of the paper, we selected a dataset with a small input size that was suitable for the size of the hardware. In this Note, we demonstrate that our approach could be employed for the classic, larger handwritten digits MNIST dataset[7].

For this purpose, we simulated, using the simulator validated in Supplementary Note 2, a two-layer $float32$ deterministic convolutional neural network followed by a two-layer (1813, 128, 10) fully connected Bayesian neural network to address the MNIST dataset. The convolution layers were using a kernel size of 5, a stride of 1, and a padding of 2. The first convolution has 1 input channel and 16 output channels, and the second one has 16 input channels and 32 output channels. The training of the Bayesian layers was calibrated on the experimental data from memristors using the technological loss described in the main article. The trained model achieved an accuracy on the MNIST test dataset of 99.2% with M=50 samples using experimental distribution achieved with two filamentary memristors per sample.

To evaluate the epistemic uncertainty performances, we incorporated into our test dataset the Kuzushiji-MNIST (KMNIST) dataset[8], as shown in Supplementary Figure 12b, which includes ten Hiragana characters in the MNIST format, and naturally provides unseen data. Supplementary Figures 12c and d depict the probability density distributions of the aleatoric and epistemic uncertainty, respectively, using the same format as Fig. 5 of the main article. Different colors are used to represent correct predictions (blue), incorrect predictions (orange), and unseen data (red). We observed that the aleatoric uncertainty is lower than 0.25 for 99% of correctly classified data points, while it exceeds 0.25 for 81% of incorrectly classified data points and unseen disease data points. Additionally, 91% of the unseen images exhibit epistemic uncertainty higher than 0.25. These results demonstrate the capability of the Bayesian neural network to identify new and unknown images in the MNIST case.

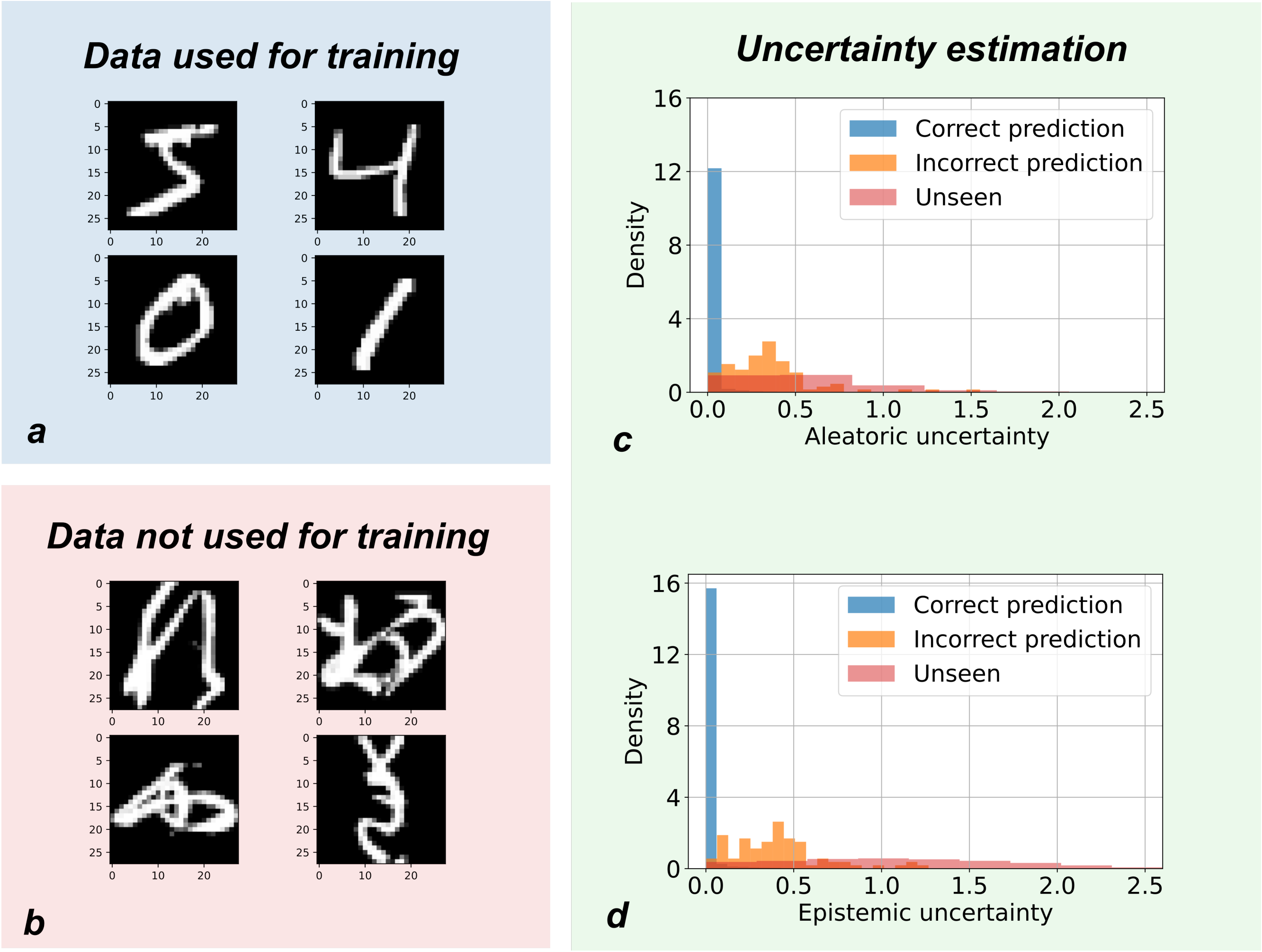

Supplementary Figure 12. Uncertainty estimation on MNIST dataset. a Images used for training. **b** Images used for out-of-distribution detection. **c** Probability density distribution of the aleatoric uncertainty for correct predictions, incorrect predictions, and unseen diseases. **d** Probability density distribution of the epistemic uncertainty for correct predictions, incorrect predictions, and unseen diseases.

Supplementary Note 12: Method validation on CIFAR datsets

In this Note, we now evaluate our approach on the CIFAR-10 image recognition dataset[9], illustrated in Supplementary Figure 13a. Our focus was specifically on evaluating our method for the last fully connected layers. For this purpose, we resized CIFAR images from 32x32 to 220x220 pixels. Additionally, we employed horizontal flipping to augment the dataset, effectively doubling its size. First, we applied the convolutional layers of a deterministic ResNet-18[10] network pre-trained on ImageNet (available in the PyTorch library) to reconstruct a features-based dataset.

Next, we used a two-layer (512, 512,10) fully connected Bayesian neural network to classify CIFAR-10 features using. The training was calibrated on the experimental data from memristors using the technological loss described in the main. The trained model achieved an accuracy of 88% with M=50 samples using experimental distribution achieved with two filamentary memristors per sample. We used the simulator validated in Supplementary Note 3,

To evaluate the epistemic uncertainty performances, we added unseen data to our test dataset. We used the data of the flower category of the CIFAR-100 dataset, shown in Supplementary Figure 13b, which is not present in CIFAR-10. Supplementary Figures 13c and d present the calculated aleatoric and epistemic uncertainties for correct predictions (blue), incorrect predictions (orange), and unseen data (red), plotted in the same format as Fig. 5 in the main body text. The aleatoric uncertainty is lower than 0.5 for 82% of all correctly classified data points, while it is higher than 0.5 for 75% of all incorrectly classified data points and unseen images. 89% of the unseen images have an epistemic uncertainty higher than 0.25. These results indicate that the Bayesian neural network can evaluate uncertainty and identify new unknown images in the complex CIFAR dataset.

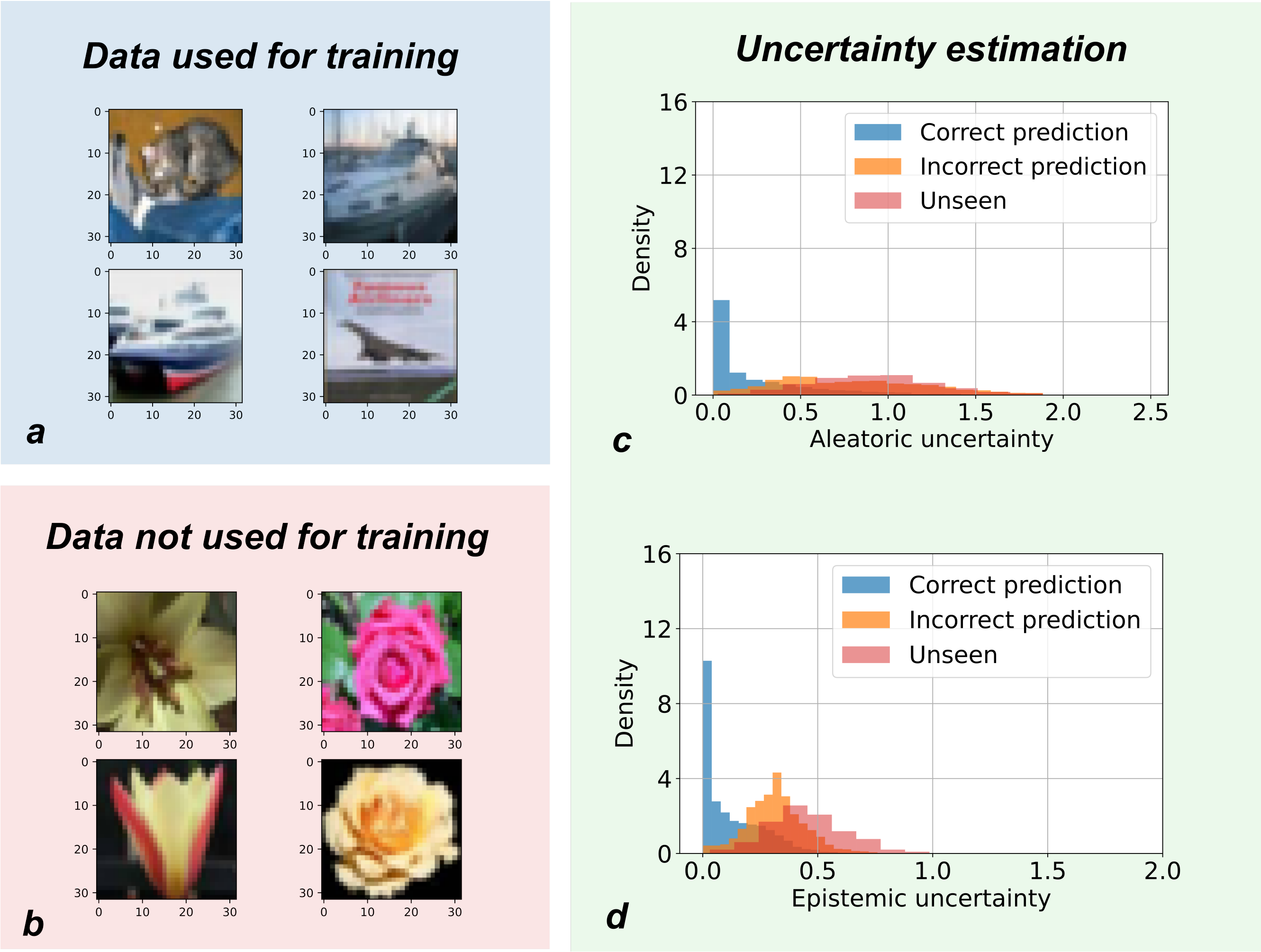

Supplementary Figure 13. Uncertainty estimation on CIFAR dataset. a Images used for training. **b** Images used for out-of-distribution detection. **c** Probability density distribution of the aleatoric uncertainty for correct predictions, incorrect predictions, and unseen diseases. **d** Probability density distribution of the epistemic uncertainty for correct predictions, incorrect predictions, and unseen diseases.

Supplementary Note 13: In-memory computing chip

This Note presents the filamentary-memristor in-memory computing dies that we used to implement a Bayesian neural network in the main article. Each die includes an array of 1,024 (32×32) filamentary memristors and the periphery circuitry to do in-memory computing. An optical microscopy photograph of such a die is shown in Fig. 2f of the main Article, and Supplementary Fig. 14 shows the simplified schematic of the die. The memristor array has one transistor-one resistor (1T1R) configuration, with word lines (WL) parallel to the source lines (SL) and orthogonal to the bit lines (BL).

The die is configured using three digital shift registers, selecting to which pad on each word line, bit line, and source line should be connected. The connection between the pads and the lines is itself realized by a collection of 64 analog multiplexers and 32 analog switches, designed using thick oxide transistors to support voltages up to five volts. The different pads V_{WL}, V_{BL}, $Vinib_{BL}$, V_{SL}, $Vinib_{SL}$ are connected to an external RIFLE NplusT engineering test system.

The memristors are programmed sequentially. The shift registers are programmed so that only the word line with the memristor to be programmed is connected to pad V_{WL}. The bit and source line shift registers are programmed into the configurations presented in Supplementary Fig. 14. Forming and SET operations use the same configuration, and RESET uses a different configuration. Once the die is configured, appropriate programming pulses are applied using the pulse generator feature of the RIFLE NplusT engineering test system. The forming operation consists in the following conditions: $V_{sl} = 0$ V, $V_{wl} = 1.6$ V, $V_{bl} \in [1.6, 4]$ V. The standard SET conditions are as follows: $V_{sl} = 0$ V, $V_{wl} \in [1.4, 2.2]$ V, $V_{bl} = 1.8$ V. The standard RESET conditions used are as follows: $V_{sl} = 2.6$ V, $V_{wl} = 4.8$ V, $V_{bl} =0$ V. The off-chip generated voltage programming pulses

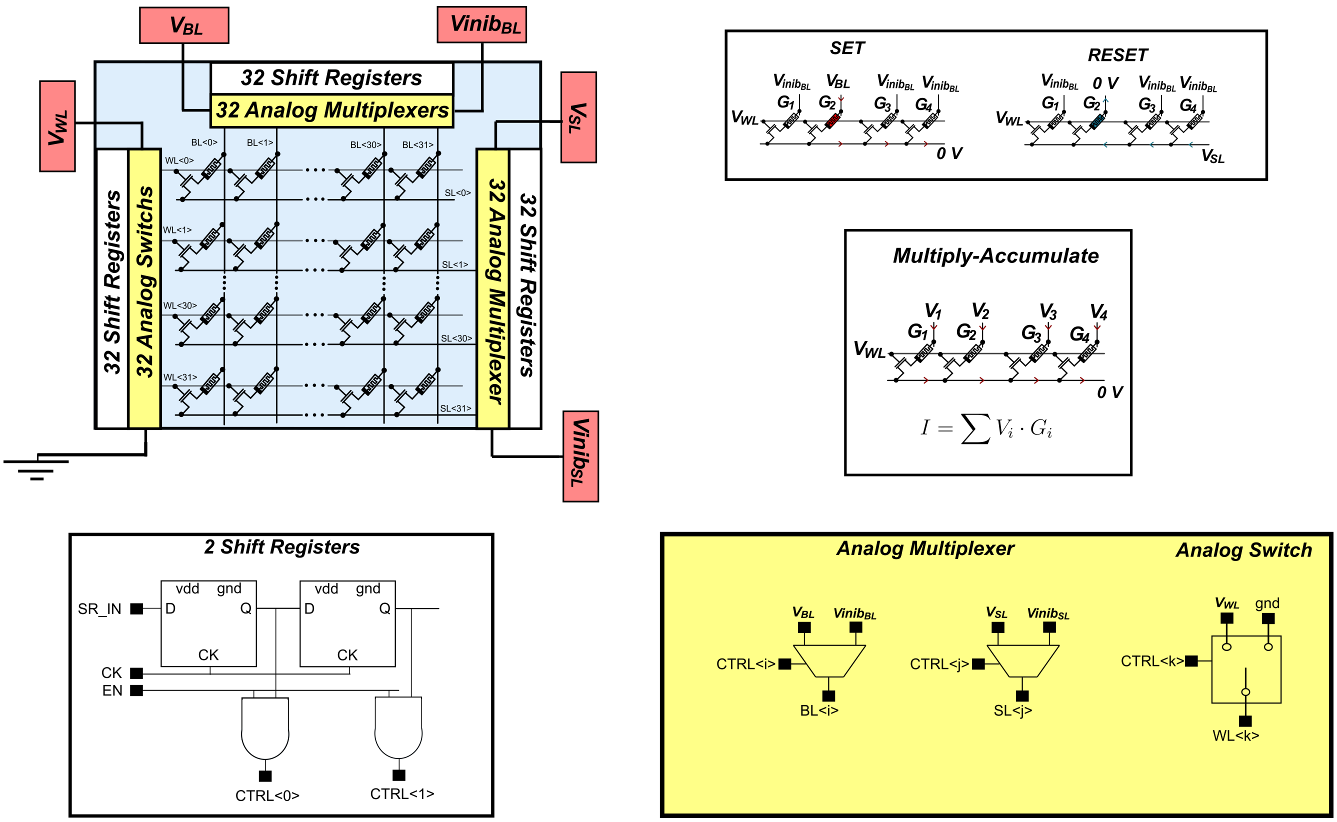

Supplementary Figure 14. Detailed version of a single crossbar array. Independent shift register chains allow the control of the bit lines (BLs), source lines (SLs), and word lines (WLs) separately. Analog multiplexers and switches drive the voltage and current necessary to operate the array.

have a pulse width of 1 μs for the SET and 100 ns for the RESET.

Inference (multiply-and-accumulate) is operated row-by-row. The shift registers are programmed so that only the word line with the memristor to be programmed is connected to pad V_{WL}. All source lines are maintained to the ground by the RIFLE NplusT test system, and the bit lines are programmed following the input of the multiply-and-accumulate. A gate voltage of 4 V is applied on the selected word line, so that the resistance of the transistor is negligible with regards to the one of the memristors. A read voltage is 0.2 V is used on the selected bit lines, small enough to not cause read disturb effects on the memristors. The current flowing through the selected source line then naturally implements the multiply-and-accumulate operation:

$$I = \sum G_i \times V_i, \tag{1}$$

where G_i are the conductance of the memristors and V_i the voltages applied on the bit lines. This current is measured and digitized by the RIFLE NplusT test system. A correction is applied to the measured current to eliminate the contribution of the parasitic series resistance fitted on experiments. To get the value of the corresponding neuron, the currents flowing through two consecutive rows of the arrays are subtracted (as each synaptic weight value is composed of two memristors, see main Article) and the activation function is applied to the result by the computer-controlling the experiment.

Supplementary Note 14: Benchmark

Alternative implementations of Bayesian neural networks with nanodevices

In the existing literature, several works have shown the potential of using memristors for Bayesian neural networks. Supplementary Table 1 lists the major characteristics of these works and provides a side-by-side comparison with our own work. Notably,

our experimental Bayesian neural network stands out as the only system capable of performing on-chip inference, thanks to a novel hardware-calibrated training algorithm based on a variation of the popular Bayes By Backprop method. In contrast, other works in the state of the art primarily relied on computer simulations calibrated using experimental data. Furthermore, we successfully detected out-of-distribution samples for the first time through the evaluation of epistemic uncertainty.

	**This work**	**Dalgaty et al.[5,11]**	**Lin et al.[12]**	**Li et al.[13]**	**Liu et al.[14]**	**Sebastaian et al.[15]**
Technology	RRAM HfO_2 PCM GST	RRAM HfO_2	RRAM TaO_x/HfO_x	RRAM	MTJ	memtransistor MoS_2
Mechanism for probabilistic distribution construction	Population of devices	Population of devices	Population of devices + read to read	Population of devices + read to read	Read to read	Cycle to cycle
Training Algorithm	Hardware calibrated training	Markov Chain Monte Carlo	Bayes By Backprop	Stochastic Weight Averaging Gaussian	Bayes By Backprop	Bayes By Backprop
Scalability	Yes	No	Yes	Yes	Yes	Yes
Inference on Chip	Yes	No	No	No	No	No
Uncertainty Evaluation	Yes	No	Yes	Yes	Yes	Yes
Anomaly Detection	Yes	No	No	No	No	No

Supplementary Table 1. Comparison of our work with approaches of the literature using memristors to store Bayesian probabilistic weights.

Comparison with the memristor-based Bayesian machine

The Bayesian neural network reported in this work uses the same hybrid hafnium-oxide memristor/CMOS process as the memristor-based Bayesian machine of Ref.[16]. However, they are very different systems. The Bayesian machine implements energy-efficient inference on Bayesian networks, whereas here, we focus on Bayesian neural networks. Beyond their Bayesian nature, these two models are extremely different. Bayesian networks are constructed using expert knowledge and are fully explainable, making them ideal for tasks such as sensor fusion. On the contrary, Bayesian neural networks are trained from the ground up and excel on more data-intensive tasks like electrocardiogram or electroencephalogram classification. Both Bayesian networks and Bayesian neural networks excel at uncertainty evaluation.

From a circuit point of view, the two designs are also strongly different, although they use a similar basic cell with two memristors. The Bayesian machine is a digital system. Using two memristors per bit cell allows reducing the amount of bit errors, and therefore to *tolerate* memristor imperfections. On the contrary, our Bayesian neural network is an analog in-memory computing system. Using two memristors per weight value allows choosing the mean value and standard deviation of synaptic weight independently, and therefore to *exploit* memristor imperfections.

Supplementary Note 15: Inference energy consumption estimates

	Wan et al. Nature 2022[17]	Cheng-Xin Xue et al. Nat. Electron. 2021[18]	Khaddam et al.IEEE JSSC 2022[19]
	HfOx/TaOx		GST
Device	RRAM	RRAM	PCM
CMOS node	130nm	22nm	14nm
Input bit width	4b	4b	8b
Output bit width	6b	11b	8b
Reported energy efficiency (TOPS/W)	16	36.61	10.5
Estimation of the energy in inference for the Bayesian hardware with N=10	1640 pJ	720 pJ	2500 pJ

Supplementary Table 2. Comparison of the energy efficiency estimated in several state-of-the-art in-memory computing circuits based on memristors and PCMs (see Methods section of the main article).

Supplementary Note 16: Flowchart of the Bayesian neural network inference experiment

This Note lists the steps required to perform the Bayesian neural network inference experiment on physical crossbar arrays, and presents step-by-step the full experiment, which includes off-chip training, the transfer of the trained neural network to memristor arrays, and on-chip inference. It is illustrated by Supplementary Fig. 15.

Off-chip training

To do the off-chip training, we need to calibrate the software to the hardware. Thus, the first step is on the experimental (probe station) side: we need to fully characterize the filamentary memristor. This characterization is performed by trying numerous programming conditions and looking at the conductance distribution. These measurements allow us to plot the technologically-plausible domain of normal distributions $\Gamma_{memristor}$. Each experimental point inside $\Gamma_{memristor}$ corresponds to two programming conditions, one for G_+ and one for G_-.

We can then prepare the training dataset and define the neural network architecture. In this work, we focus on a fully connected Bayesian neural network with 32 input neurons, 16 hidden neurons, and nine output neurons. The next step is to train the Bayesian neural network, i.e., identify the mean value and standard deviation of all synapses of the network. We do a first training using variational inference, without supplementary hardware constraint on the synaptic weights. Based on these training results, we compute the scaling factor γ (see Methods of the main Article, equation (4)). This factor allows us to map the weight in software to the conductance in hardware. At this point, we define the Technological loss $-\log(U_\Gamma(\theta))$ (see main Article, equations (1-2)), and we retrain the Bayesian neural network from scratch incorporating this technological loss.

Crossbar programming (transfer to hardware)

From the off-chip training, we get the targeted mean conductance and standard deviation for each synapse. Each synapse is programmed using two memristors, in each of the M memory arrays. From the measurements of $\Gamma_{memristor}$, we get a set of the programming conditions for the two memristors that can implement the normal law the closest to the target for each synapse. (As described in the Methods, we use the KL divergence to find these two best programming conditions.) This way of programming the memristor is immune to their imperfections, as the imperfections are fully incorporated in the normal laws of the memristors.

The first layer of our neural network uses an array of 32×16 synapses, and thus 32×32 programming conditions. Therefore, we use a full array (Supplementary Note 5) to implement one sample of the first layer. The second layer of our neural network is

only 32×9 synapses, and thus 16×18 programming conditions. Thus we can implement two samples using an array. Therefore, to have M samples representing our Bayesian neural network we need to program $1.5M$ arrays.

On-chip inference

Once the memristor arrays have been programmed, we validate our programmed neural network with a test data set using on-chip inference. The inputs, which are converted to binary patterns (see Methods), are applied to the bit lines of each of the arrays representing the first layer of the Bayesian neural network, implementing naturally a multiply-and-accumulate function (see Supplementary Note 5). With the resulting source lines currents, we get the activations (see Methods). Then we apply binary patterns representing the activations to the bit lines of each of the arrays representing the second layer of the Bayesian neural network. With the resulting source lines currents, we compute the predictions (see Methods). Note that all the computations made externally (activation and prediction) for this phase are due to our demonstrator limitation. In a final system, all these operations will be performed on-chip.

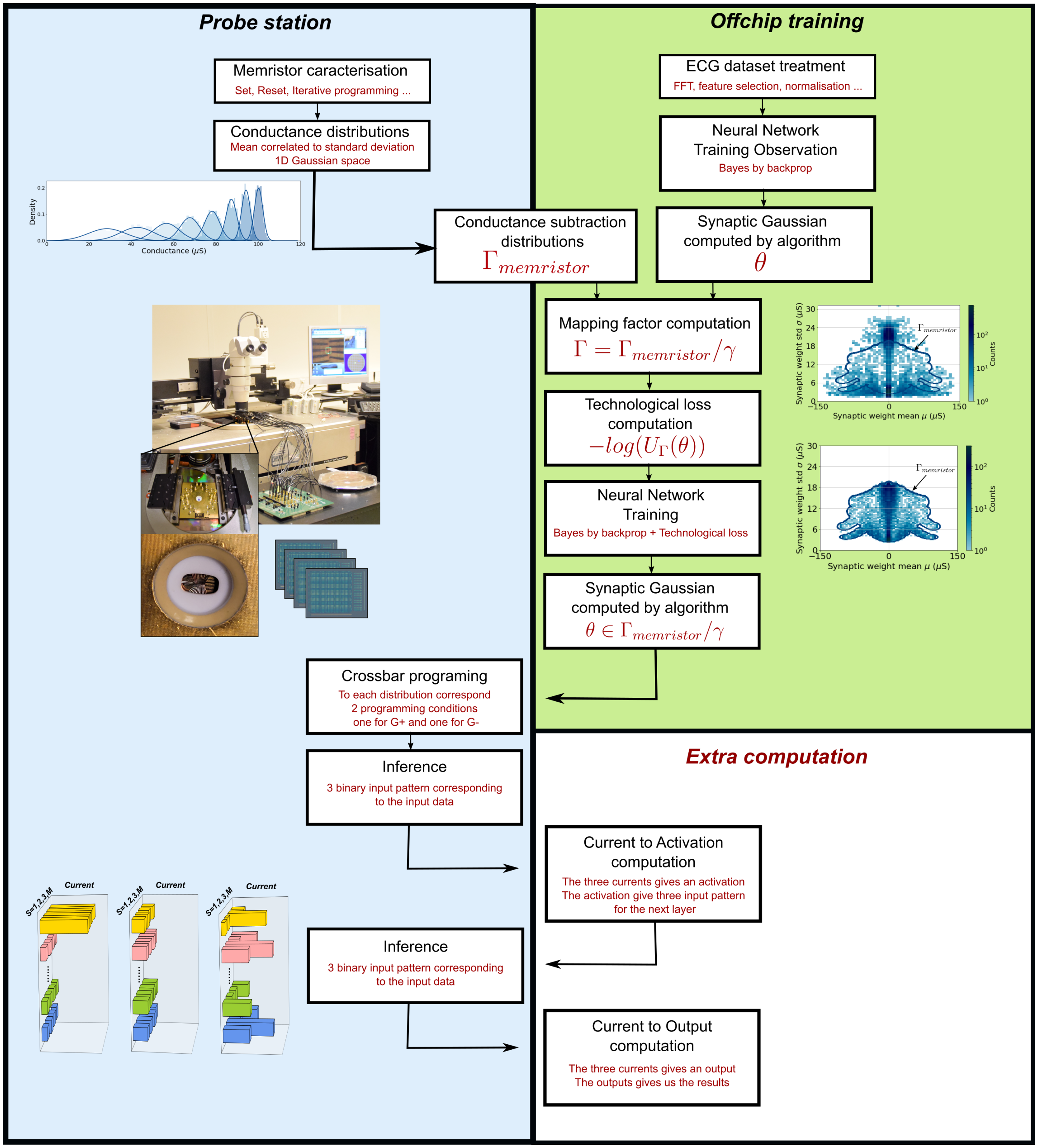

Supplementary Figure 15. Flow chart of the Bayesian neural network inference experiment on physical crossbar arrays.

Supplementary References

1. Trabelsi, A. *et al.* Frequency modulation of conductance level in pcm device for neuromorphic applications. In *ESSCIRC 2022-IEEE 48th European Solid State Circuits Conference (ESSCIRC)*, 129–132 (IEEE, 2022).

2. Joshi, V. *et al.* Accurate deep neural network inference using computational phase-change memory. *Nat. communications* **11**, 1–13 (2020).

3. Esmanhotto, E. *et al.* High-density 3d monolithically integrated multiple 1t1r multi-level-cell for neural networks. In *2020 IEEE International Electron Devices Meeting (IEDM)*, 36–5 (IEEE, 2020).

4. Esmanhotto, E. *et al.* Experimental demonstration of multilevel resistive random access memory programming for up to two months stable neural networks inference accuracy. *Adv. Intell. Syst.* 2200145 (2022).

5. Dalgaty, T., Esmanhotto, E., Castellani, N., Querlioz, D. & Vianello, E. Ex situ transfer of bayesian neural networks to resistive memory-based inference hardware. *Adv. Intell. Syst.* **3**, 2000103 (2021).

6. Jospin, L. V., Buntine, W., Boussaid, F., Laga, H. & Bennamoun, M. Hands-on bayesian neural networks–a tutorial for deep learning users. *arXiv preprint arXiv:2007.06823* (2020).

7. LeCun, Y., Bottou, L., Bengio, Y. & Haffner, P. Gradient-based learning applied to document recognition. *Proc. IEEE* **86**, 2278–2324 (1998).

8. Clanuwat, T. *et al.* Deep learning for classical japanese literature (2018). cs.CV/1812.01718.

9. Krizhevsky, A. & Hinton, G. Learning multiple layers of features from tiny images. Tech. Rep. 0, University of Toronto, Toronto, Ontario (2009).

10. He, K., Zhang, X., Ren, S. & Sun, J. Deep residual learning for image recognition. In *Proceedings of the IEEE conference on computer vision and pattern recognition*, 770–778 (2016).

11. Dalgaty, T., Vianello, E. & Querlioz, D. Harnessing intrinsic memristor randomness with bayesian neural networks. In *2021 International Conference on IC Design and Technology (ICICDT)*, 1–4 (IEEE, 2021).

12. Lin, Y. *et al.* Bayesian neural network realization by exploiting inherent stochastic characteristics of analog rram. In *2019 IEEE International Electron Devices Meeting (IEDM)*, 14–6 (IEEE, 2019).

13. Li, X. *et al.* Enabling high-quality uncertainty quantification in a pim designed for bayesian neural network. In *2022 IEEE International Symposium on High-Performance Computer Architecture (HPCA)*, 1043–1055 (IEEE, 2022).

14. Liu, S. *et al.* Bayesian neural networks using magnetic tunnel junction-based probabilistic in-memory computing. *Front. Nanotechnol.* 78 (2022).

15. Sebastian, A. *et al.* Two-dimensional materials-based probabilistic synapses and reconfigurable neurons for measuring inference uncertainty using bayesian neural networks. *Nat. communications* **13**, 1–10 (2022).

16. Harabi, K.-E. *et al.* A memristor-based bayesian machine. *Nat. Electron.* 1–12 (2022).

17. Wan, W. *et al.* A compute-in-memory chip based on resistive random-access memory. *Nature* **608**, 504–512 (2022).

18. Xue, C.-X. *et al.* A cmos-integrated compute-in-memory macro based on resistive random-access memory for ai edge devices. *Nat. Electron.* **4**, 81–90 (2021).

19. Khaddam-Aljameh, R. *et al.* Hermes-core—a 1.59-tops/mm 2 pcm on 14-nm cmos in-memory compute core using 300-ps/lsb linearized cco-based adcs. *IEEE J. Solid-State Circuits* **57**, 1027–1038 (2022).
